# Comparative Effectiveness of Combining MTX with Biologic Drug Therapy Versus Either MTX or Biologics Alone for Early Rheumatoid Arthritis in Adults: a Systematic Review and Network Meta-analysis

**DOI:** 10.1007/s11606-019-05230-0

**Published:** 2019-08-06

**Authors:** Katrina E. Donahue, Elizabeth R. Schulman, Gerald Gartlehner, Beth L. Jonas, Emmanuel Coker-Schwimmer, Sheila V. Patel, Rachel Palmieri Weber, Carla M. Bann, Meera Viswanathan

**Affiliations:** 1 University of North Carolina Department of Family Medicine, Chapel Hill, NC USA; 2 Cecil G Sheps Center for Health Services Research, Chapel Hill, NC USA; 3 Hospital for Special Surgery, New York, NY USA; 4 Department of Medicine, Division of Rheumatology, Allergy, and Immunology, University of North Carolina, Chapel Hill, NC USA; 5 RTI International, Research Triangle Park, NC USA; 6 Department for Evidence-based Medicine and Clinical Epidemiology, Danube University, Krems, Austria

## Abstract

**Background:**

Comparative effectiveness of early rheumatoid arthritis (RA) treatments remains uncertain.

**Purpose:**

Compare benefits and harms of biologic drug therapies for adults with early RA within 1 year of diagnosis.

**Data Sources:**

English language articles from the 2012 review to October 2017 identified through MEDLINE, Cochrane Library and International Pharmaceutical Abstracts, gray literature, expert recommendations, reference lists of published literature, and supplemental evidence data requests.

**Study Selection:**

Two persons independently selected studies based on predefined inclusion criteria.

**Data Extraction:**

One reviewer extracted data; a second reviewer checked accuracy. Two independent reviewers assigned risk of bias ratings.

**Data Synthesis:**

We identified 22 eligible studies with 9934 participants. Combination therapy with tumor necrosis factor (TNF) or non-TNF biologics plus methotrexate (MTX) improved disease control, remission, and functional capacity compared with monotherapy of either MTX or a biologic. Network meta-analyses found higher ACR50 response (50% improvement) for combination therapy of biologic plus MTX than for MTX monotherapy (relative risk range 1.20 [95% confidence interval (CI), 1.04 to 1.38] to 1.57 [95% CI, 1.30 to 1.88]). No significant differences emerged between treatment discontinuation rates because of adverse events or serious adverse events. Subgroup data (disease activity, prior therapy, demographics, serious conditions) were limited.

**Limitations:**

Trials enrolled almost exclusively selected populations with high disease activity. Network meta-analyses were derived from indirect comparisons relative to MTX due to the dearth of head-to-head studies comparing interventions. No eligible data on biosimilars were found.

**Conclusions:**

Qualitative and network meta-analyses suggest that the combination of MTX with TNF or non-TNF biologics reduces disease activity and improves remission when compared with MTX monotherapy. Overall adverse event and discontinuation rates were similar between treatment groups.

**Registration:**

PROSPERO (available at http://www.crd.york.ac.uk/PROSPERO/display_record.php?ID=CRD42017079260).

## INTRODUCTION

Rheumatoid arthritis (RA) is an autoimmune systemic inflammatory disease affecting more than 1 million Americans and characterized by synovial inflammation, which can lead to progressive bone erosion, joint damage, and disability.^1^ For patients with early RA (≤ 1 year of disease),^2^ guidelines recommend early treatment with the goal of remission or low disease activity.^3, 4^ Available therapies for RA include corticosteroids, conventional synthetic disease-modifying antirheumatic drugs (csDMARDs), tumor necrosis factor (TNF) and non-TNF biologics, targeted synthetic DMARDs (tsDMARDs), and biosimilars. Over the past two decades, biologics have become an important treatment option for established RA. However, clinicians face the challenge around biologic use in early RA.

Biologics commonly used for RA treatment include TNF biologics (adalimumab, certolizumab pegol, etanercept, golimumab, infliximab) and non-TNF biologics (abatacept, rituximab, tocilizumab, and sarilumab). Experts and guideline groups support using csDMARDs, often methotrexate (MTX), as the first line-therapy.^3, 4^ Despite recommendations, advocates encourage early biologic use to induce remission.^5, 6^

In a 2012 systematic review, evidence comparing early RA treatment options was limited.^7^ No studies investigated efficacy, effectiveness, and harms among subgroup populations. Recently, information from clinical trials of four biosimilar drugs (ADA-atto, IFX-dyyb, IFX-abda, ETN-szzs), a tsDMARD (tofacitinib), and one non-TNF biologic (sarilumab) have become available. Additionally, studies continue to be published on established therapies. Given this uncertainty, the Agency of Healthcare Research and Quality (AHRQ) and the Patient Centered Outcomes Research Institute (PCORI) commissioned a systematic review to compare effectiveness and harms of RA drugs in patients with early RA. This paper focuses on comparisons of benefits and harms of treatments in early RA involving biologics.

## METHODS

The full technical report describes the study methods in detail^8^ and the protocol was registered at PROSPERO (http://www.crd.york.ac.uk/PROSPERO/display_record.php?ID=CRD42017079260). In a comprehensive synthesis of the evidence, we included data from studies dating back to June 2006, identified in the 2012 review on this topic^7^ and through an updated literature search.

### Data Sources

A professional research librarian searched MEDLINE, Cochrane Library, Embase, and International Pharmaceutical Abstracts from January 2011 to October 5, 2017. We re-reviewed studies included in the 2012 review,^7^ supplemental evidence (data received through the AHRQ Web site and a Federal Register notice), and reference lists of included studies and recent reviews. We also searched the following sources for unpublished studies: ClinicalTrials.gov, World Health Organization International Clinical Trials Registry Platform, and New York Academy of Medicine’s Grey Literature Index.

### Study Selection

We included study populations defined as early RA by the authors if the diagnosis was ≤ 1 year in the past (Table 1 presents inclusion and exclusion criteria). Two reviewers independently reviewed titles and abstracts using abstrackr^9^ and full-text articles for eligibility. To assess efficacy regarding disease activity, response, remission, radiographic progression, and functional capacity, we included head-to-head controlled trials and prospective cohort studies comparing any of the therapies. In addition, we included placebo- and MTX-controlled trials for network meta-analyses (NWMA). For adverse events, we abstracted data on overall adverse events, overall study discontinuation, discontinuation attributed to adverse events or toxicity, patient adherence, and any serious adverse events as defined by the FDA.^10^ For specific adverse events (that were not serious adverse events), we focused on those most commonly reported according to their FDA-approved labels.

**Table 1:** Eligibility Criteria

PICOTS	Inclusion	Exclusion
Population	Adult outpatients 18 years of age or older with a diagnosis of early RA, defined as 1 year or less from disease diagnosis; studies with mixed populations if > 50% of study populations had an early RA diagnosis Subpopulations by age, sex or gender, race or ethnicity, disease activity, prior therapies, concomitant therapies, and other serious conditions	Adolescents and adult patients with disease greater than 1 year from diagnosis; inpatients
Intervention	TNF biologics: adalimumab, certolizumab pegol, etanercept, golimumab, infliximab Non-TNF biologics: abatacept, rituximab, sarilumab, tocilizumab	Anakinra is excluded because, although it is approved for RA, clinically it is not used for this population^61^; non-biologic therapies for RA
Comparator	For head-to-head RCTs, head-to-head nRCTs, and prospective, controlled cohort studies: any active intervention listed above For additional observational studies of harms and among subgroups: any active intervention listed above For double-blinded, placebo-controlled trials for network meta-analysis: placebo	All other comparisons, including active interventions not listed above; no comparator; dose-ranging studies that are not comparing two different interventions
Outcomes	Disease activity, response, remission, radiographic joint damage Functional capacity, quality of life, patient-reported outcomes Overall risk of harms, overall discontinuation, discontinuation because of adverse effects, risk of serious adverse effects, specific adverse effects*, patient adherence	All other outcomes not listed
Timing	At least 3 months of treatment	< 3 months treatment
Settings	Primary, secondary, and tertiary care centers treating outpatients	Facilities treating inpatients only
Country setting	Any geographic area	None
Study designs	Study designs include head-to head RCTs and nRCTs; prospective, controlled cohort studies (*N* > 100); double-blinded, placebo-controlled trials for network meta-analysis; and SRs only to identify additional references For studies of harms—i.e., overall and among subgroups, study designs also included any other controlled observational study (e.g., cohort, case-control) (*N* > 100)	All other designs not listed
Publication language	English	Languages other than English

*FDA* US Food and Drug Administration; *KQ* key question; *N* number; *nRCT* nonrandomized controlled trial; *PICOTS* population, intervention/exposure, comparator, outcomes, time frames, country settings, study design; *RA* rheumatoid arthritis; *RCT* randomized controlled trial; *SR* systematic review; *TNF* tumor necrosis factor

*Most commonly reported according to their FDA-approved labels—rash, upper respiratory infection, nausea, pruritus, headache, diarrhea, dizziness, abdominal pain, bronchitis, leukopenia, and injection site reactions

### Data Extraction and Risk of Bias Assessment

Trained reviewers abstracted each study using a structured, pilot-tested form and a senior reviewer evaluated accuracy. To assess the risk of bias (ROB), we adapted the Cochrane Risk of Bias tool^11^ for randomized controlled trials (RCTs) and used the Risk of Bias in Non-randomised Studies of Interventions (ROBINS-I) tool^12^ for nonrandomized controlled studies.

### Data Synthesis and Analyses

We planned to conduct pairwise analysis when possible and NWMAs to estimate the indirect treatment effects. Criteria for eligible studies for NWMA included^1^ no failed prior treatment attempt with MTX^2^, treatment doses within FDA-approved ranges^3^, 12-month follow-up, and^4^ double-blinded RCTs of low or medium ROB. Head-to-head and placebo-controlled RCTs were eligible for NWMA; however, we did not find any eligible placebo-controlled trials in a population with early RA. We considered NWMA for American College of Rheumatology 50% improvement (ACR50), Disease Activity Score (DAS) remission, radiographic joint damage, all study discontinuations, and discontinuations attributed to adverse events.

**Figure 1:**
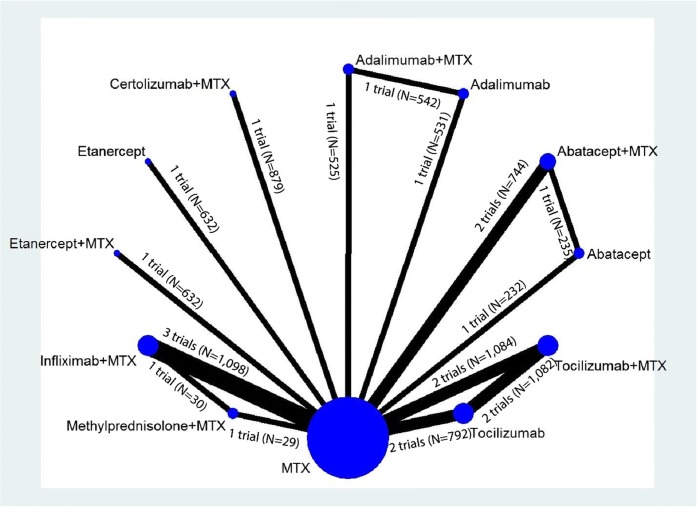
Network diagram for network meta-analysis of ACR50 response rates. MTX, methotrexate; N, number of patients**.**

**Figure 2:**
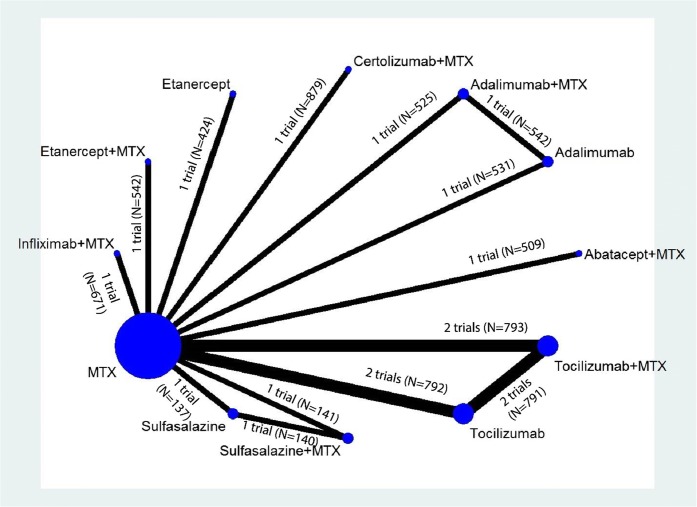
Network diagram for network meta-analysis of change from baseline in radiographic joint damage score. MTX, methotrexate; N, number of patients**.**

We ran NWMAs using a multivariate, random effects meta-regression model with restricted maximum likelihood for variance estimation.^13^ Models were fit using the Stata “network” package^14^ an updated versions of the “mymeta” package which accounts for multi-arm trials. The network structure for outcomes was mostly “star-shaped,” indicating a dearth of head-to-head studies directly comparing interventions (see Figs. 1 and 2, low strength of evidence). Most effect estimates, therefore, were derived from indirect comparisons relative to MTX rather than mixed treatment comparisons. For closed loops, we tested the transitivity assumption by examining loop-specific consistency between direct and indirect effects using network side splits and global consistency by comparing a model assuming consistency with a model not assuming consistency (i.e., inconsistency model). When the global Wald test indicated no significant differences between the consistency and inconsistency models,^15^ and no significant differences in estimates based on side splits, we presented consistency model estimates.

### Strength of Evidence

We evaluated strength of evidence for each comparison based on the guidance established for AHRQ’s EPC Program as high, moderate, and low or insufficient.^16^ We graded strength of evidence for the following outcomes: disease activity, response, radiographic joint damage, functional capacity, discontinuation because of adverse events, and serious adverse events.

### Role of the Funding Source

This topic was nominated and funded by PCORI in partnership with AHRQ. The AHRQ Contracting Officer’s Representative and PCORI program officers provided comments on the protocol and full evidence report. Neither PCORI nor AHRQ directly participated in literature searches; study eligibility criteria determination; data analysis or interpretation; or preparation, review, or manuscript approval for publication.

## RESULTS

### Characteristics of Reviewed Studies

**Figure 3:**
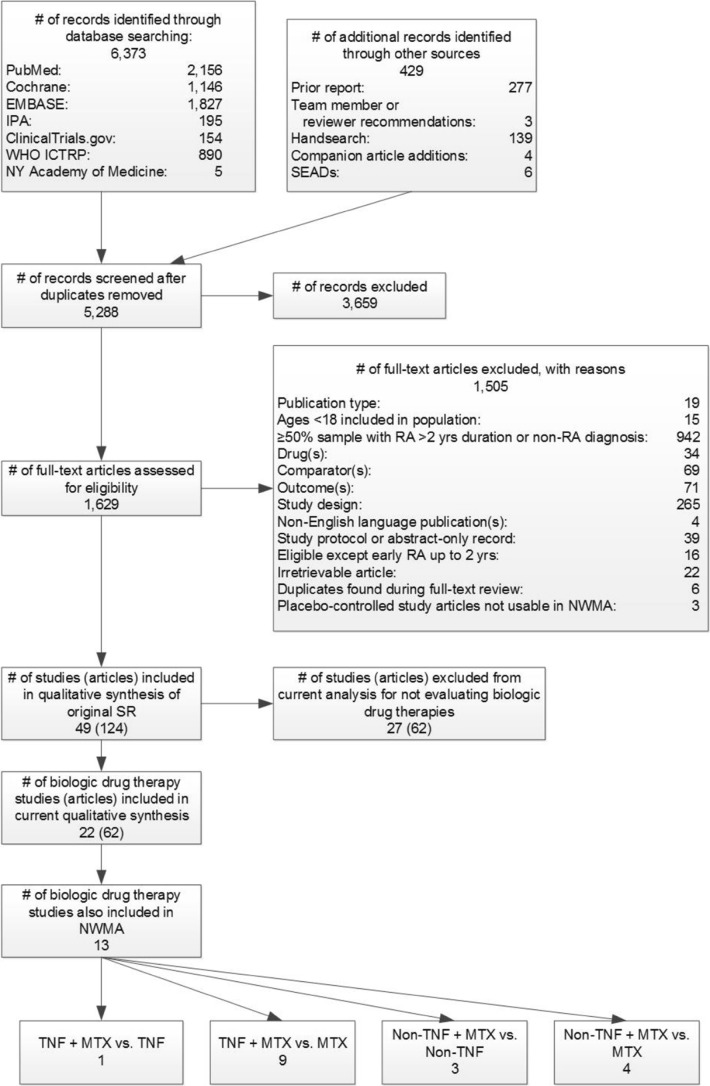
Summary of literature search flow and yield for early rheumatoid arthritis. IPA, International Pharmaceutical Abstracts; MTX, methotrexate; NWMA, network meta-analysis; NY, New York; RA, rheumatoid arthritis; SEADs, supplemental evidence and data; TNF, tumor necrosis factor; vs., versus; WHO ICTRP, World Health Organization International Clinical Trials Registry Platform; yrs, years**.**

We identified 6373 citations from electronic searches and 429 from other sources (Fig. 3). We were unable to use pairwise meta-analyses due to a lack of head-to-head studies. In this paper, we report results from trials of biologic comparisons only. For these comparisons, we found 22 RCTs with low or medium risk of bias (Table 2). We included 13 studies in our NWMA.

**Table 2:** Characteristics of Included Trials

Characteristics	
Studies (articles)	22 (61)
Patients	9,934
Range of % female	53 to 81
Age: range of means	46 to 57
Risk of bias (*N* studies)*	Low: 4 Medium: 17 High: 7
Study duration	1 to 2 years
*N* studies reporting on benefits (articles)	22 (61)
*N* studies reporting on harms (articles)	22 (59)
*N* studies reporting on subgroup effects (articles)	4 (17)

*N* number

*Some studies received more than one risk of bias rating because we assigned different ratings to specific outcomes reported by the same study. For this reason, the *N*’s of studies with different ratings will not add up to the total of 22 studies included in this paper.

Study durations ranged from 6 months to 2 years. Over half of the study populations were women (range 53 to 81%) with mean ages ranging from 46 to 57 years. Included studies almost exclusively enrolled patients with high disease activity at baseline as measured by mean or median Disease Activity Score (DAS) 28 scores (range of mean scores 3.6 to 7.1). Among studies reporting MTX use, 18 studies (82%) enrolled MTX-naïve patient samples; the remaining 3 studies enrolled patients with prior csDMARD use (including MTX). Most trials used ACR response, disease activity scores to measure clinical improvement, and Sharp or Sharp/van der Heijde scores to measure radiographic progression of the disease. Trials examining function or quality of life most commonly used the Health Assessment Questionnaire (HAQ) or Medical Outcomes Study Short Form 36 (SF-36). Harms studies generally described overall withdrawals, withdrawals due to adverse events, and specific adverse events including the most commonly occurring across all eligible drugs according to their FDA-approved labels. The majority (*N* = 21, 95%) were at least partially industry funded. Table 3 summarizes main findings and the strength of evidence. The remainder of results are organized such that we present evidence on the combination of biologics with MTX compared first to biologic monotherapy and second to MTX monotherapy for both TNF and non-TNF biologics. NWMA, when available, follows the comparisons.

**Table 3:** Summary of Findings About Benefits and Harms of Treatments for Early Rheumatoid Arthritis with Strength of Evidence Grades

Key comparisons		Efficacy Strength of evidence		Harms Strength of evidence	
Treatment types	Specific treatments	Rating	Explanation	Rating	Explanation
TNF biologics vs. MTX	ADA + MTX vs. ADA vs. MTX	Moderate	ACR response and remission significantly higher, radiographic progression less, and functional capacity significantly improved with ADA + MTX vs. ADA or with ADA vs. MTX.^17^	Moderate	No significant differences in discontinuation because of adverse events or serious adverse events for ADA + MTX vs. ADA or for ADA vs. MTX^17^
Non-TNF biologics vs. MTX	ABA + MTX vs. ABA vs. MTX	Low	No significant differences in ACR response^50, 53^ or remission^50^ for ABA + MTX vs. ABA or for ABA vs. MTX	Low	No significant differences in discontinuation because of adverse events or serious adverse events for ABA + MTX vs. ABA or for ABA vs. MTX^50^
	TCZ + MTX vs. TCZ or TCZ vs. MTX	Low	Remission significantly higher for TCZ + MTX vs. TCZ and TCZ vs. MTX^51, 52^	Moderate	No significant differences in discontinuation because of adverse effects or serious adverse events for TCZ + MTX vs. TCZ or for TCZ vs. MTX^51, 52^
		Insufficient	Functional capacity and disease activity^51, 52^		
	ADA + MTX vs. MTX	Moderate	Functional capacity significantly improved for ADA + MTX vs. MTX^17, 22, 24, 26, 33, 62^	Low	No significant differences in discontinuation because of adverse events for ADA + MTX vs. MTX^17, 22, 24, 26, 33, 62^
		Low	ACR response significantly higher with ADA + MTX vs. MTX^17, 22, 24, 26, 33, 62^	Low	No significant differences in serious adverse events for ADA + MTX vs. MTX^17, 22, 24, 26, 33, 62^
		Low	Remission significantly higher with ADA + MTX vs. MTX^17, 22, 24, 26, 33, 62^		
		Low	Radiographic progression less with ADA + MTX vs. MTX^17^		
TNF biologic + MTX vs. MTX monotherapy	CZP + MTX vs. MTX	Low	ACR response^36, 63^ significantly higher and radiographic progression^20^ less for CZP + MTX vs. MTX	Low	No significant differences in discontinuation because of adverse effects or serious adverse events^20^
		Low	Remission significantly higher and functional capacity improved for CZP + MTX vs. MTX^20^		
	ETN + MTX or ETN vs. MTX	Moderate	ACR response significantly higher and radiographic progression less for ETN + MTX and ETN vs. MTX^37, 38^	Low	No significant differences in discontinuation because of adverse effects or serious adverse events^37, 38^
		Low	Remission rates significantly higher for ETN + MTX and ETN vs. MTX^37, 38^		
		Low	Functional capacity mixed for ETN + MTX and ETN vs. MTX^37, 38^		
	IFX + MTX vs. MTX	Low	Remission rates^45, 46^ significantly higher and functional capacity^45, 46^ greater for IFX + MTX vs. MTX	Low	No significant differences in discontinuation because of adverse effects or serious adverse events^45^
		Insufficient	Disease activity^45–47^ and radiographic progression^45, 46^ for IFX + MTX vs. MTX		
TNF biologic vs. csDMARD combination therapy (e.g., triple therapy)	IFX + MTX vs. MTX + SSZ + HCQ	Low	ACR response significantly higher for IFX + MTX vs. MTX + SSZ+ HCQ^64^	Low	No significant differences in discontinuation because of adverse effects or serious adverse events.^64^
	IFX + MTX + SSZ + HCQ+ PRED vs. MTX + SSZ + HCQ + PRED	Low	No significant differences in ACR response, radiographic progression, or remission for IFX + MTX + SSZ + HCQ + PRED vs. MTX + SSZ + HCQ + PRED^65^	Low	No significant differences in discontinuation because of adverse effects or serious adverse events^65^
		Low	No significant differences in functional capacity for IFX + MTX + SSZ + HCQ + PRED vs. MTX + SSZ + HCQ + PRED^65^		
Non-TNF biologic vs. MTX monotherapy	ABA + MTX vs. MTX	Moderate	Disease activity significantly improved and remission rates higher for ABA + MTX vs. MTX^50, 53^	Low	No significant differences in discontinuation because of adverse effects or serious adverse events^53^
		Low	Radiographic progression significantly less for ABA + MTX vs. MTX^53^		
		Low	Functional capacity mixed for ABA + MTX vs. MTX^50, 53^		
	RIT + MTX vs. MTX	Moderate	Disease activity significantly improved and radiographic progression less for RIT + MTX vs. MTX^57^	Moderate	No significant differences in discontinuation because of adverse effects or serious adverse events^57^
		Moderate	Remission rates significantly higher for RIT + MTX vs. MTX^57^		
		Moderate	Functional capacity significantly improved for RIT + MTX vs. MTX^57^		
	TCZ + MTX vs. MTX	Moderate	Radiographic progression less for TCZ + MTX vs. MTX^51, 52^	Moderate	No significant differences in discontinuation because of adverse effects or serious adverse events^51, 52^
		Low	Remission significantly higher for TCZ + MTX vs. MTX^51, 52^		
		Insufficient	Disease activity and functional capacity for TCZ + MTX vs. MTX^51, 52^		
TNF vs. non-TNF biologics	RIT vs. ADA or ETN	Low	Functional capacity significantly improved for RIT vs. ADA or ETN^66^	Insufficient	Discontinuation because of adverse effects or serious adverse events^66^
		Insufficient	Disease activity or remission for RIT vs. ADA or ETN^66^		

*ABA* abatacept, *ACR* American College of Rheumatology, *ADA* adalimumab, *csDMARD* conventional synthetic DMARD, *CZP* certolizumab pegol, *DAS* Disease Activity Score, *DMARD* disease-modifying antirheumatic drug, *ETN* etanercept, *HCQ* hydroxychloroquine, *IFX* infliximab, *MTX* methotrexate, *PRED* prednisone, *RIT* rituximab, *TCZ* tocilizumab, *TNF* tumor necrosis factor, *tsDMARD* targeted synthetic DMARD, *vs.* versus

### TNF Biologics

#### TNF Biologic Plus Methotrexate Versus TNF Biologic Monotherapy

One RCT of adalimumab (ADA) provided evidence for direct comparison of a TNF biologic plus MTX with TNF biologic monotherapy.^17^ The NWMA provided some information for ETN as noted below. No comparisons were available for certolizumab pegol (CZP), golimumab (GOL), or infliximab (IFX).

##### Adalimumab

The PREMIER study^17^ (*N* = 799) compared ADA (40 mg biweekly) plus MTX (20 mg/week) with ADA monotherapy (or MTX monotherapy further described below) in MTX-naïve patients with early aggressive RA.^17^ ADA plus MTX had significantly higher ACR50 response (59% vs. 37%, respectively, *p* < 0.001), smaller radiographic changes (modified Sharp/van der Heijde score [mTSS], 1.9 vs. 5.5, respectively; *p* < 0.001), and higher remission rates (DAS28 < 2.6; 49% vs. 25%, respectively, *p* < 0.001) than ADA monotherapy at 2 years. Additionally, the combination therapy group achieved greater improvement in functional capacity than the ADA monotherapy group (HAQ-DI mean change, − 1.1 vs. − 0.8, respectively; *p* = 0.0002). During the 10-year open-label extension,^18^ patients taking ADA plus MTX had significantly less radiographic progression than those on ADA monotherapy, but results were limited by a 34% attrition rate.

**Figure 4:**
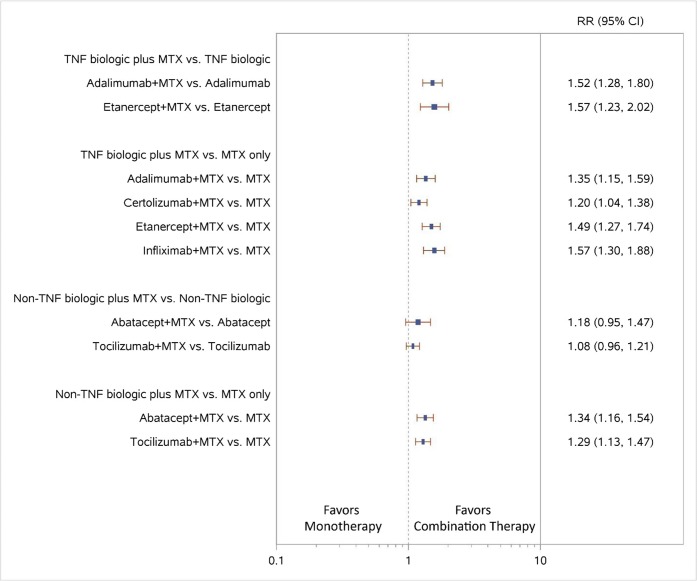
Forest plot for network meta-analysis (low SOE grades for all NWMA effect estimates) of biologic plus MTX vs. biologic or MTX only: ACR50 response rates. 95% CI, 95% confidence interval; MTX, methotrexate; RR, relative risk; TNF, tumor necrosis factor; vs., versus**.**

**Figure 5:**
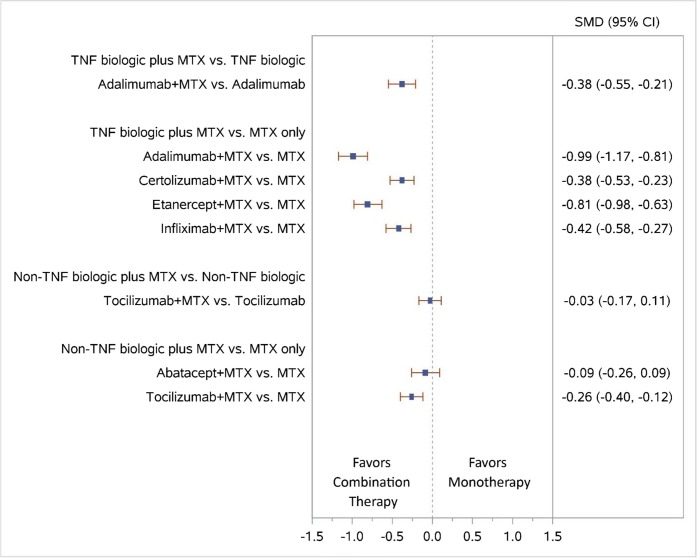
Forest plot for network meta-analysis (low SOE grades for all NWMA effect estimates) of biologic plus MTX vs. biologic MTX only: change from baseline in radiographic joint damage score. 95% CI, 95% confidence interval; MTX, methotrexate; SMD, standardized mean difference (mean difference divided by standard deviation); TNF, tumor necrosis factor; vs., versus**.**

Results of the NWMA also favored the combination of ADA plus MTX versus ADA monotherapy for higher ACR50 response (relative risk [RR], 1.52; 95% confidence interval [CI], 1.28 to 1.80) (Fig. 4) and less radiographic progression (standardized mean difference [SMD], − 0.38; 95% CI, − 0.55 to − 0.21) (Fig. 5).

##### Etanercept (ETN)

No study examined ETN plus MTX compared with ETN monotherapy directly; NWMA favored the combination of ETN plus MTX over ETN monotherapy for higher ACR50 response (RR, 1.57; 95% CI, 1.23 to 2.02) (Fig. 4). NWMA examining ETN plus MTX versus ETN monotherapy found no significant differences in all discontinuations or discontinuations due to adverse events (data not shown).

#### TNF Biologic Plus Methotrexate Versus Methotrexate Monotherapy

Thirteen RCTs compared a TNF biologic plus MTX with MTX monotherapy. Overall, the TNF biologics plus MTX had smaller radiographic changes and higher remission rates than MTX monotherapy. NWMA found lower overall discontinuation rates for combination therapy consisting of TNF biologics (specifically, ADA, CZP, and ETN, but not IFX) plus MTX than MTX monotherapy (range of RR, 0.64 [95% CI, 0.53 to 0.78] to 0.66 [95% CI, 0.43 to 1.00]) (data not shown). However, neither serious adverse events nor discontinuations because of adverse events differed between the groups.

##### Adalimumab

Five RCTs examined the combination of ADA (40 mg biweekly) plus MTX (ranging from 8 to 20 mg/week) with MTX monotherapy over 26 weeks to 2 years.^17–35^ Results were consistent: four trials showed improvements in disease activity and functional improvement, and five trials showed smaller radiographic changes for the combination of ADA plus MTX; two trials showed no significant differences but trended in favor of combination therapy. The trials showing differences were conducted over a shorter period (26 weeks). NWMA found higher ACR50 responses and less radiographic progression for ADA plus MTX combination therapy than for MTX monotherapy (RR, 1.35; 95% CI, 1.15 to 1.59, and SMD, − 0.99; 95% CI, − 1.17 to − 0.81, respectively) (Figs. 4 and 5).

##### Certolizumab Pegol

Two RCTs examined the combination of CZP plus MTX versus MTX monotherapy in MTX-naïve patients.^20, 36^ One 24-week Japanese trial^20^ and one 52-week multinational trial^36^ randomized patients with early RA and poor prognostic factors (high anti-cyclic citrullinated peptide antibody, positive RF, or bony erosions) to CZP plus MTX or to MTX monotherapy. Both trials reported significantly higher DAS28-ESR remission rates (score < 2.6^36^ or not defined^20^) and functional capacity and significantly lower radiographic progression among patients receiving combination therapy than among patients receiving MTX monotherapy.

In the NWMA, higher ACR50 response rates and less radiographic progression were noted for CZP plus MTX combination therapy than MTX monotherapy (RR, 1.20; 95% CI, 1.04 to 1.38, and SMD, − 0.38; 95% CI, − 0.53 to − 0.23, respectively) (Figs. 4 and 5).

##### Etanercept

Three trials compared ETN plus MTX with MTX monotherapy.^37–39^ The first trial included 542 patients with early RA followed over 2 years.^37, 40–44^ Patients in the ETN plus MTX group had a significantly higher ACR50 response (70.7% vs. 49.0%, *p* < 0.001) and greater improvement in functional capacity (HAQ mean change − 1.02 vs. − 0.72, *p* < 0.0001) than MTX monotherapy at 52 weeks. Remission was also significantly higher in the ETN plus MTX group (DAS44 remission < 1.6; 51.3% vs. 27.8%, *p* < 0.0001). The second trial found no significant difference in ACR20 response rates, radiographic changes, or physical function at 12 months.^36^ The third trial^39^ did not find any significant differences in DAS28 between groups but was of shorter duration (24 weeks) and smaller sample size (*n* = 26).

In the NWMA, higher ACR50 response rates and less radiographic progression were also noted for ETN plus MTX combination therapy than MTX monotherapy (RR, 1.49; 95% CI, 1.27 to 1.74, and SMD, − 0.81; 95% CI, − 0.98 to − 0.63, respectively) (Figs. 4 and 5).

##### Infliximab

Three trials examined the combination of IFX plus MTX compared with MTX monotherapy in MTX-naïve patients.^45–47^ The largest trial (*n* = 1049) compared the efficacy of initiating two different combinations of IFX (3 mg/kg or 6 mg/kg) plus MTX (20 mg/week) with MTX monotherapy over 54 weeks^45, 48, 49^ and found improved ACR response rates and HAQ scores for both IFX plus MTX combination therapy groups compared with MTX monotherapy (ACR50: 45.6% vs. 50.4% vs. 31.1%, *p* < 0.001, respectively; patients with HAQ increase ≥ 0.22 units from baseline: 76.0%, 75.5%, 65.2%, *p* < 0.004, respectively). Patients treated with IFX plus MTX also had higher rates of remission (DAS28-ESR < 2.6: 21.3% for IFX combination therapy groups vs. 12.3% for MTX only, *p* < 0.001)^49^ and less radiographic progression (modified SHS change 0.4 to 0.5 for IFX combination therapy groups vs. 3.7 for MTX only, *p* < 0.001).^45^ The smaller trials found improved^46^ or trending^47^ ACR50 responses in favor of IFX combination therapy at 54 weeks among patients receiving IFX plus MTX combination therapy.

In the NWMA, IFX plus MTX combination therapy led to higher ACR50 response rates and less radiographic progression than MTX monotherapy (RR, 1.57; 95% CI, 1.30 to 1.88, and SMD, − 0.42; 95% CI, − 0.58 to − 0.27, respectively) (Figs. 4 and 5).

### Non-TNF Biologics

#### Non-TNF Biologic Plus Methotrexate Versus Non-TNF Biologic Monotherapy

##### Abatacept (ABA)

One RCT, the multinational AVERT trial (*n* = 351), compared the combination of ABA (125 mg/week) plus MTX (7.5 mg/week) with ABA monotherapy.^50^ This double-blind RCT compared treatments over 1 year; at year 2, patients with DAS28-CRP < 3.2 were tapered off treatment. If patients experienced an RA flare by month 15, they were given ABA plus MTX. No significant differences were noted for ABA plus MTX versus ABA monotherapy for ACR50 response, remission (DAS28-CRP < 2.6), or functional capacity.

##### Tocilizumab (TCZ)

Two RCTs assessed differences in efficacy between a TCZ plus MTX combination and TCZ monotherapy in MTX-naïve populations.^51, 52^ The FUNCTION tria1^51^ examined TCZ plus MTX combination therapy over 1 year in 1162 patients with early aggressive RA (moderate to severe active RA classified by ACR criteria). After 1 year, 49% in the TCZ (8 mg/kg/month) plus MTX (10–30 mg/week) combination, and 39.4% in the TCZ monotherapy group achieved remission (DAS28-ESR < 2.6) (*p* < 0.001). The U-Act-Early trial^52^ examined 317 patients with early RA over 2 years. Patients were randomized to TCZ (8 mg/kg/month) plus MTX (10–30 mg/week), or TCZ monotherapy. At 2 years, there were no differences in remission for TCZ plus MTX versus TCZ monotherapy (DAS28 < 2.6); 86% vs. 88%). Both trials reported less radiographic progression with TCZ plus MTX than with MTX monotherapy.

#### Non-TNF Biologic Plus Methotrexate Versus Methotrexate Monotherapy

##### Abatacept

The AGREE trial was a multinational trial of 509 early RA patients (98% MTX naïve) with poor prognostic factors comparing ABA plus MTX with MTX monotherapy over 2 years.^53–56^ After 1 year, the ABA plus MTX group had significantly higher ACR50 response and greater functional benefit than the MTX monotherapy group (ACR50: 57.4% vs. 42.3%, respectively, *p* < 0.001; HAQ-DI % change of > 0.3 units: 71.9% vs. 62.1%, respectively, *p* = 0.024). The ABA plus MTX group also had significantly higher remission rates (DAS28-CRP < 2.6: 41.4% vs. 23.3%, *p* < 0.001) and less mean radiographic changes (Genant-modified Sharp score: 0.63 vs. 1.06, *p* = 0.040) than the MTX monotherapy group. Less radiographic progression was noted at 2 years for the ABA plus MTX group compared with the MTX monotherapy group.^55^

The multinational AVERT study (*n* = 351) compared the combination of ABA plus MTX with MTX monotherapy.^50^ At 1-year, patients in the ABA plus MTX group had significantly higher remission rates than the MTX monotherapy comparison group (DAS28-CRP < 2.6: 60.9% vs. 45.2%, respectively, *p* = 0.010). Remission rates remained higher for ABA plus MTX than for the MTX monotherapy group following treatment withdrawal at 18 months (DAS28-CRP < 2.6: 14.8% vs. 7.8%, respectively, *p* = 0.045). Overall, ABA plus MTX had smaller radiographic changes and higher remission rates than MTX monotherapy.

The NWMA found significant differences in ACR50 response when comparing ABA plus MTX with MTX monotherapy (RR, 1.34; 95% CI, 1.16 to 1.54), consistent with results from the AGREE and AVERT trials (Fig. 4). The combination of ABA plus MTX had numerically less radiographic progression than MTX monotherapy, but the difference was not significant (SMD, − 0.09; 95% CI, − 0.26 to 0.09) (Fig. 5).

In NWMA, there was no difference in overall discontinuation between ABA plus MTX and MTX alone, though ABA plus MTX had fewer discontinuations due to adverse events (RR, 0.49, 95% CI, 0.28 to 0.86) (data not shown).

##### Rituximab

One trial^57–59^ (*n* = 755) randomized MTX-naïve patients to rituximab (RIT) (1 g days 1 and 15) plus MTX (7.5–20 mg/week), RIT (500 mg days 1 and 15) plus MTX, or MTX monotherapy over 52 weeks. Both RIT plus MTX groups had significantly improved disease activity (DAS28: 43%, 40%, 20%, respectively, *p* < 0.001) and remission rates (DAS28-ESR < 2.6: 31%, 25%, 13%, respectively, *p* < 0.0010) and less radiographic change (0.36, 0.65, 1.08, respectively, *p* < 0.001) compared with MTX monotherapy. Overall, RIT plus MTX had smaller radiographic changes and higher remission rates than MTX monotherapy. Functional capacity (measured by HAQ-DI decrease > 0.22) improved more in both of the RIT plus MTX groups than in the MTX monotherapy group (HAQ response, 88% and 87% vs. 77%; *p* < 0.05). Discontinuation because of adverse events and serious adverse events did not differ across groups.

##### Tocilizumab

Two RCTs, the FUNCTION trial^51^ (*N* = 1162) and the U-Act-Early trial^52^ (*N* = 317), both previously described in the “Non-TNF Biologic Plus Methotrexate Versus Non-TNF Biologic Monotherapy” section, assessed differences in efficacy between TCZ plus MTX and MTX monotherapy in MTX-naïve populations. In both trials, TCZ plus MTX combination therapy led to smaller radiographic changes and higher remission rates (DAS28-ESR < 2.6: 49% vs. 19.5%, *p* < 0.001) than MTX monotherapy after 1 to 2 years. Both trials demonstrated greater functional capacity in the combination group than the MTX monotherapy group. Overall discontinuation rates, discontinuation because of adverse events, and serious adverse events did not differ across groups.

### Subgroups

Only three RCTs compared drug therapies among different subgroups defined by demographics, disease activity, or coexisting conditions.^33, 38, 45^ We could not draw any conclusions about response rates or serious adverse events between older and younger patients or between people with different levels of disease activity.

## DISCUSSION

Although several biologic agents are available, head-to-head evidence remains limited. Combination therapy with TNF or non-TNF biologics plus MTX resulted in improved disease control, DAS-defined remission, and functional capacity compared with monotherapy of either MTX or a biologic. Network meta-analyses (NWMAs) found higher ACR50 response for combination therapy of biologic plus MTX than MTX monotherapy. The results of comparative NWMA for overall discontinuation and discontinuation attributed to adverse events had confidence intervals too wide to support firm conclusions. Subgroup data were limited.

Eligible early RA studies almost exclusively included patients with high disease activity. In contrast, patients with early RA may present in a clinical setting with varying levels of severity. Patients with early RA enrolled in trials consist of highly selected individuals.^60^ Although the evidence for the effectiveness of biologics plus MTX in early RA is favorable, it is not the standard of care for several reasons. First, some data indicate that certain patients will do well on MTX monotherapy, but no information is available about how to identify or predict who these patients will be.^33, 38, 45^ Second, many insurers require MTX failure as a prerequisite to add a biologic. Third, some patients may be wary of a combination therapy approach in early disease (e.g., cost, side effects, injections).

Several limitations of our review should be considered. No consensus exists on defining early RA. For this review, we defined populations with early RA as having a diagnosed disease duration of 1 year or less and included mixed population studies if > 50% of the study populations had an early RA diagnosis. Patients described in this way may have had longer symptoms. Although existing evidence of biologics in combination with MTX shows that this regimen can improve disease activity, we do not know whether starting biologic treatment rather than MTX improves long-term prognosis. Because of a lack of head-to-head trials, we often relied on NWMA to estimate the comparative effectiveness of interventions of interest for treating patients with early RA. NWMAs are an important analytic tool in the absence of direct head-to-head evidence, but also have limitations; thus, we graded them as low strength of evidence. NWMAs often yield estimates with wide confidence intervals that encompass clinically relevant benefits or harms for both drugs (or combination therapies) being compared. The network geometry was mostly star-shaped with very few closed loops, which limited the number of tests we could use to assess transitivity and consistency. The FDA has approved several biosimilars, but there were no eligible studies of biosimilars.

Future research directions include comparisons of patients with different degrees of disease activity or poor prognostic factors and longer-term effects. Data are needed for examination of biosimilars. Studies with longer treatment periods and follow-up of 5 years or longer would provide better information on long-term effectiveness, adherence, and adverse events. They would also yield insights as to whether starting with a biologic improves long-term prognosis of RA.

Analyses of subpopulations based on age and coexisting medical conditions would also be helpful for clinicians and patients newly diagnosed with RA. Currently, treatment selection based on benefits and harms is difficult in these populations. Additionally, patient-centered research is needed with appropriate use of patient-reported outcomes and other patient-generated health data so that results are truly reflective of patient preferences and desires.

In conclusion, for patients with early RA and almost exclusively high disease activity, qualitative data and NWMAs suggest that the combination of a TNF or non-TNF biologic with MTX improves disease activity and DAS-defined remission when compared with either biologic or MTX monotherapy.
